# A compendium of *DIS3* mutations and associated transcriptional signatures in plasma cell dyscrasias

**DOI:** 10.18632/oncotarget.4674

**Published:** 2015-07-20

**Authors:** Marta Lionetti, Marzia Barbieri, Katia Todoerti, Luca Agnelli, Sonia Fabris, Giovanni Tonon, Simona Segalla, Ingrid Cifola, Eva Pinatel, Pierfrancesco Tassone, Pellegrino Musto, Luca Baldini, Antonino Neri

**Affiliations:** ^1^ Department of Clinical Sciences and Community Health, University of Milano, Milan, Italy; ^2^ Hematology Unit, Fondazione IRCCS Ca’ Granda, Ospedale Maggiore Policlinico, Milan, Italy; ^3^ Laboratory of Pre-Clinical and Translational Research, IRCCS-CROB, Referral Cancer Center of Basilicata, Rionero in Vulture (PZ), Italy; ^4^ Functional Genomics of Cancer Unit, Division of Experimental Oncology, IRCCS San Raffaele Scientific Institute, Milan, Italy; ^5^ Institute for Biomedical Technologies, National Research Council, Milan, Italy; ^6^ Department of Experimental and Clinical Medicine, Magna Graecia University, Catanzaro, Italy

**Keywords:** multiple myeloma, plasma cell leukemia, DIS3, next-generation sequencing

## Abstract

DIS3 is a catalytic subunit of the human exosome complex, containing exonucleolytic (RNB) and endonucleolytic (PIN) domains, recently found mutated in multiple myeloma (MM), a clinically and genetically heterogeneous form of plasma cell (PC) dyscrasia. We analyzed by next-generation sequencing (NGS) the *DIS3* PIN and RNB domains in purified bone marrow PCs from 164 representative patients, including 130 cases with MM, 24 with primary PC leukemia and 10 with secondary PC leukemia. *DIS3* mutations were found respectively in 18.5%, 25% and 30% of cases. Identified variants were predominantly missense mutations localized in the RNB domain, and were often detected at low allele frequency. *DIS3* mutations were preferentially carried by *IGH*-translocated/nonhyperdiploid patients. Sequential analysis at diagnosis and relapse in a subset of cases highlighted some instances of increasing *DIS3* mutation burden during disease progression. NGS also revealed that the majority of *DIS3* variants in mutated cases were comparably detectable at transcriptional level. Furthermore, gene expression profiling analysis in *DIS3*-mutated patients identified a transcriptional signature suggestive for impaired RNA exosome function. In conclusion, these data further support the pathological relevance of *DIS3* mutations in plasma cell dyscrasias and suggest that *DIS3* may represent a potential tumor suppressor gene in such disorders.

## INTRODUCTION

Multiple myeloma (MM) is a fatal malignancy of bone marrow (BM) plasma cells (PCs) whose pathogenesis is only partially understood. PC dyscrasias show a wide clinical presentation ranging from the presumed pre-malignant condition of monoclonal gammopathy of undetermined significance (MGUS) to smoldering MM (SMM), symptomatic MM, and extra-medullary MM or plasma cell leukemia (PCL) [1]. PCL can be distinguished into primary PCL (pPCL), originating *de novo* without any prior history of MM, or secondary PCL (sPCL), arising from a preexisting myeloma tumor that eventually progressed to the leukemic phase [2].

At the genetic level, MM is characterized by both numerical and structural chromosomal alterations [1] alongside somatic gene mutations, as recently emerged from whole genome/exome sequencing analyses [3–6]. In fact, the general scenario arising from such studies indicated a considerable number of non-synonymous variants per patient, few recurrently mutated genes of probable pathogenetic significance, and a heterogeneous subclonal structure at the time of diagnosis [3–6]. Among the recurrently mutated genes with putative pathogenetic role, the most frequent were *KRAS, NRAS, TP53, BRAF, TRAF3, FAM46C* and *DIS3* [3, 4, 6–8].

The *DIS3* gene maps at 13q22.1 and encodes the catalytic subunit of the human RNA exosome complex, prominently participating to RNA processing and turnover [9]. DIS3 catalytic activity is governed by the endoribonucleolytic (PIN) and the 3′–5′ exoribonucleolyitic (RNB) domains. It is indispensable for survival in vertebrates [10]. Somatic mutations and altered expression have been reported in several cancer types (COSMIC catalogue, Release v71, http://cancer.sanger.ac.uk/cancergenome/projects/cosmic/) [11, 12]. Concerning MM, *DIS3* was reported as mutated on average in 11% of patients [4, 6, 7, 13]. Some *DIS3* mutations identified in MM showed to interfere with its exonucleolytic activity, causing aberrant RNA metabolism and slower proliferation rate in HEK293-derived human cell lines [10]. Furthermore, very recently we demonstrated that DIS3 facilitates the maturation of the tumor suppressor *let-7* miRNAs by reducing in the cytoplasm the RNA stability of the pluripotency factor LIN28B, an inhibitor of *let-7* processing. Through the reduction of mature *let-7*, DIS3 enhances the translation of *let-7* targets such as MYC and RAS leading to enhanced tumorigenesis [14].

Here, we assessed by means of next-generation sequencing (NGS) the mutational status of *DIS3* gene in a large and representative series of patients at different stages of plasma cell dyscrasia, and related it with other biological and clinical features; furthermore, we analyzed for the first time the gene expression pattern characterizing *DIS3*-mutated MM patients.

## RESULTS

### Assessment of *DIS3* mutations in PC dyscrasias

To estimate the frequency of *DIS3* mutations in different phases of plasma cell dyscrasia, 164 cases (including 130 MM, 24 pPCL and 10 sPCL patients) and 20 human myeloma cell lines (HMCLs) were subjected to deep sequencing of the two functional domains of the gene, namely PIN and RNB domains.

The median depth of coverage was 245x (range: 64–1160x). After exclusion of intronic, synonymous and germline variants, deep sequencing of *DIS3* revealed 32 different coding somatic mutations globally targeting 27 genic positions (Table 1). The presence of each non-synonymous single nucleotide variant (SNV) or indel was validated in an independent PCR product, analyzed by conventional sequencing whenever the sensitivity of the Sanger method (i.e. about 10% in our experimental conditions) was consistent with the variant allele frequency (VAF), or by an additional ultra-deep pyrosequencing run (median depth of coverage = 1110x) in case of variants at low allele frequency. At DNA level, 29 out of 32 variants (90.6%) were SNVs, all resulting in missense mutations but one (73355968C > T, M1_V16del), which replaces one base within the first coding ATG, thus relocating the translation start site 16 codons downstream without altering the reading frame. The remaining three variants (9.4%) were indels: two involved a single different nucleotide causing a frameshift, while the other was a 27bp-deletion resulting in an in-frame deletion of nine amino acids (L48_A56del). Nonsense mutations were not observed. Two of the identified variants were exclusively carried by two HMCLs (see below).

**Table 1:** Summary of *DIS3* non-synonymous/indel variants identified by NGS in the present dataset

Variant*	AA change	dbSNP ID (v142)/COSMIC ID (v71)	Previously reported in MM	Mutated samples (VAF)				
**73346338C > T**	D488N	COSM158635	[6, 13]	KMS27 (49.7%)	MM-343 (46.2%)	PCL-041 (45.7%)	MM-398 (3.8%)	MM-042 (4.6%)
**73336064C > T**	R780K	COSM329311	[4, 6, 7, 13]	PCL-019 (47.7%)	PCL-042 (45.9%)	MM-281 (36.8%)		
**73355093T > C**	T93A	/	/	PCL-021 (100%)	MM-213 (56.6%)			
**73335837G > A**	R820W	rs372878316/COSM3469577	/	MM-445 (98.4%)	MM-207 (2.1%)			
**73335930G > A**	R789W	/	/	MM-335 (94.2%)	PCL-011 (91.1%)			
**73336078A > C**	F775L	/	[6]	MM-036 (92.6%)	MM-414 (4.7%)			
**73336064C > G**	R780T	/	[6]	PCL-035 (45.%)	MM-381 (16.4%)			
**73352393A > C**	V171G	/	http://www.keatslab.org	KMS26 (100%)				
**73336112T > C**	H764R	/	/	PCL-036 (97.6%)				
**73337707G > T**	A670D	/	/	PCL-001 (97%)				
**73355010G > C**	F120L	/	/	MM-123 (95.3%)				
**73355048G > A**	R108C	/	/	MM-424 (94.3%)				
**73355008T > G**	Y121S	/	[19]	OPM2 (91.8%)				
**73355018T > C**	K118E	/	/	PCL-015 (90.1%)				
**73355804_73355830del**	L48_A56del	/	/	MM-340 (54.9%)				
**73355968C > T**	M1_V16del	/	/	MM-464 (51%)				
**73336151G > T**	A751D	/	/	MM-340 (45.7%)				
**73346340T > A**	D487V	/	/	MM-317 (40.3%)				
**73346341C > G**	D487H	/	/	MM-372 (35.5%)				
**73345240G > A**	S550F	/	/	MM-386 (35.1%)				
**73342930C > T**	E626K	/	/	PCL-042 (33.2%)				
**73346400C > T**	R467Q	rs201674523/COSM1944518	[6]	MM-263 (31.1%)				
**73336113G > A**	H764Y	/	/	MM-207 (24.6%)				
**73346400del**	R467Qfs*4	/	/	MM-263 (5.9%)				
**73355110T > C**	N87S	/	/	MM-279 (2.9%)				
**73337650C > T**	R689Q	COSM1367629	[6]	MM-340 (2.2%)				
**73336113G > C**	H764D	/	/	MM-207 (1.9%)				
**73336077T > G**	T776P	/	/	MM-207 (0.8%)				
**73343049_73343050insA**	A586Vfs*7	/	/	MM-055 (0.8%)				
**73355891T > C**	D27G	/	/	MM-143 (0.6%)				
**73345094T > C**	H568R	/	/	MM-310 (0.5%)				
**73345097T > C**	N567S	/	/	MM-150 (0.5%)				

* Genomic positions based on hg19.

**Figure 1:**
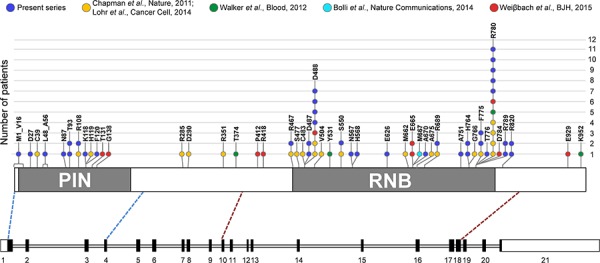
Frequency of SNVs and in-frame deletions in *DIS3* coding region in MM, including information from literature In the lower part of the figure, a schematic diagram of the *DIS3* gene is reported, based on exon-intron organization of the longer transcript variant, NM_014953.4. Exons are numbered under the boxes, filled with black in the coding region. Exons sequenced in this study (i.e., exons 1–4 and exons 10–18) encompass respectively the entire PIN (cd09862, amino acids 9–197) and RNB domain (pfam00773, amino acids 467–791), plus portions of the flanking regions, as indicated by the blue and red dashed lines connecting the diagram of the gene to the scheme of the DIS3 protein, in the upper part of the figure. Here, somatic mutations identified in the present series and reported in the main MM datasets [3, 4, 6, 7, 13] are depicted.

As for primary patients, 21 out of 30 mutations (70%) affected the RNB domain, while only eight variants (8/30, 26.7%) were located in the PIN domain. The remaining SNV mapped in a region just downstream of the RNB domain. Overall, the most affected residue was the arginine at position 780 (R780), replaced by a lysine or by a threonine in three and two cases, respectively (Figure 1). This is in line with what reported in previous MM sequencing studies, where this amino acid position was already indicated as the major *DIS3* mutational hotspot [4, 6, 7, 13]. The arginine at position 780 was found involved in RNA binding function [15]. Both R780K and R780T substitutions are recurrent in MM patients (of our and previously published datasets [6, 7, 13]), and the former was demonstrated to cause significant aberrations of DIS3 exonucleolytic activity and growth inhibition in yeast and human cellular models [10]. The second most affected residue in our dataset was the aspartic acid at position 488 (D488), involved in magnesium ion binding at the active site [15]. This residue was mutated in four cases, all carrying the D488N substitution, which also represents the globally most frequent variant in our series. Of note, D488 represents the second most affected residue in *DIS3-* mutated MM patients when combining the results of the previous studies [3, 4, 6, 7, 13]. Interestingly, the adjacent conserved aspartic acid at position 487 is equally essential for magnesium binding, and when substituted with asparagine (D487N) it was reported to completely abolish DIS3 exonuclease activity in yeast and human cellular models, causing molecular phenotypes comparable to the *DIS3*^R780K^ mutant [10]. Notably, two missense variants involving D487 residue were detected in our dataset (D487V and D487H, each in one patient). These mutations were not previously reported in MM. Other recurrently mutated positions in our series were T93, H764, F775, R789 and R820, each targeted in two samples by the same substitution, except for H764 (reported to be involved in RNA binding [15]) showing different amino acid changes. Notably, the majority of these recurrently altered residues represent novel *DIS3* mutations in MM. In fact only the variant F775L has already been reported in another series [6]. The 17 remaining genic positions were found privately mutated in our cohort. Some of them have been already reported as altered in MM (R108, R467, S550) [6] or in other cancer types (R820, COSM3469577); among the mutated residues never described before, A751 is involved in RNA binding [15]. Similar to other studies [3, 4, 6, 7], we did not find any mutation involving the residue E665, proposed as mutational hotspot by WeiΔbach and colleagues based on their sequencing results [13].

Notably, four patients carried multiple *DIS3* mutations; only in two of these cases (MM-340 and PCL-042) two variants were both at high allele frequency, while in the remaining samples only one of the mutations was above the detection limit of Sanger method, accompanied by other variants at low allele frequency. In particular, PCL-042 harbored the variants R780K and E626K, both in the RNB domain, in 46% and 33% of reads, respectively, and MM-340 carried a nine amino acid in-frame deletion in the PIN domain (L48_A56del) and a missense substitution in the RNB domain (A751D) in 55% and 46% of reads, respectively.

Beyond the aforementioned R780 and D487 residues, whose importance for the catalytic activity of the protein has been functionally proven, a potential detrimental effect on DIS3 nucleolytic activity can be suggested also for many of the other mutations we identified here, on the basis of their recurrence (as described above), and/or the high conservation of affected amino acid residues, and/or the functional predictions returned by different algorithms. In fact, from the alignment of human DIS3 protein sequence to mouse and yeast paralogs and to *E.coli* RNases II and R sequences, we found that 13 of the 24 genic positions targeted by mutations resulting in amino acid substitutions or in-frame deletions in our primary patients encoded amino acid residues conserved in all the analyzed species (Supplementary Figure S1). Furthermore, 21 out of the 26 missense mutations were suggested to be functionally relevant according to five different prediction tools (Supplementary Table S1).

Regarding *DIS3* mutations found in the cell lines, three out of the 20 analyzed HMCLs were mutated for this gene. In particular, KMS27 carried the aforementioned D488N hotspot mutation, while OPM2 and KMS26 cells harbored the variants Y121S and V171G, respectively. Both these substitutions were located in the PIN domain and were absent in primary tumors of both our and other series; only the V171G involved a highly conserved residue and was suggested to be functionally relevant by all the bioinformatics predictors. Our data concerning HMCLs are concordant with those reported in the MM Cell Line Characterization Project (available at http://www.keatslab.org/data-repository).

### *DIS3* mutations in different phases and molecular subtypes of PC dyscrasia

*DIS3* mutations globally affected 33 patients (33/164, 20.1%): specifically, 24 MMs at diagnosis (24/130, 18.5%), six primary PCLs (6/24, 25%), and three secondary PCLs (3/10, 30%). The most relevant molecular features of the 33 mutated cases are reported in Supplementary Table S2.

We next asked whether there were any relationships with cytogenetic lesions. We observed a positive association between *DIS3* mutations and IGH@ translocations (*P* value = 0.0046), particularly with the t(11;14) (*P* value = 0.0198), and a lower occurrence of hyperdiploidy among *DIS3-* mutated cases (*P* value = 0.025). Conversely, we were unable to find any association with 17p deletion, 1q gain or 1p loss (Table 2). Although not significantly associated with 13q14 deletion, *DIS3* mutations occurred in several cases carrying this chromosomal alteration. Specifically, 19 of the 33 (57.6%) *DIS3*-mutated patients harbored del(13q14). For eight of them, genome-wide DNA profiling data were also available [16, 17], indicating that in all but one case (PCL-019) the chromosomal deletion included the *DIS3 locus*. Interestingly, VAFs around 100% were exclusively observed in a fraction of patients (10/19) with 13q deletion (and in all likelihood with heterozygous deletion of the *DIS3 locus*). In the other cases, i.e. the remaining del(13q14) samples (9/19) and all the 14 patients disomic for chromosome 13, the VAFs we found were indicative of mutations occurring in hemizygosis in tumor subclones or carried in heterozygosis either by all or by a fraction of myelomatous cells (Supplementary Figure S2).

**Table 2:** Clinical and molecular characteristics of the 164 MM/PCL patients analyzed for *DIS3* mutations

Characteristic	All patients (*n* = 164)		*DIS3* wild type (*n* = 131)		*DIS3*-mutated (*n* = 33)		*P* value^a^
	**N**	**%**	***n***	**%**	***n***	**%**	
MM	130	*79*	106	*81*	24	*73*	n.s.
pPCL	24	*15*	18	*14*	6	*18*	
sPCL	10	*6*	7	*5*	3	*9*	
del (13q)	77	*47*	58	*45*	19	*58*	n.s.
chr 13 disomic patients	86	*53*	72	*55*	14	*42*	
del (17p)	17	*10.5*	15	*12*	2	*6*	n.s.
17p disomic patients	145	*89.5*	114	*88*	31	*94*	
1q gain	66	*43*	49	*39*	17	*57*	n.s.
1q disomic patients	89	*57*	76	*61*	13	*43*	
1p loss	18	*13*	16	*14*	2	*7*	n.s.
1p disomic patients	123	*87*	97	*86*	26	*93*	
*IGH* translocation	81	*50*	58	*44*	23	*74*	0.0046
no *IGH* translocation	81	*50*	73	*56*	8	*26*	
hyperdiploid	49	*33*	45	*37*	4	*14*	0.0250
non-hyperdiploid	101	*67*	77	*63*	24	*86*	

a Significance was assessed by Freeman-Halton extension of Fisher's exact test for disease type, and by Fisher's exact test for all other variables. *n.s*.: not significant.

**Figure 2:**
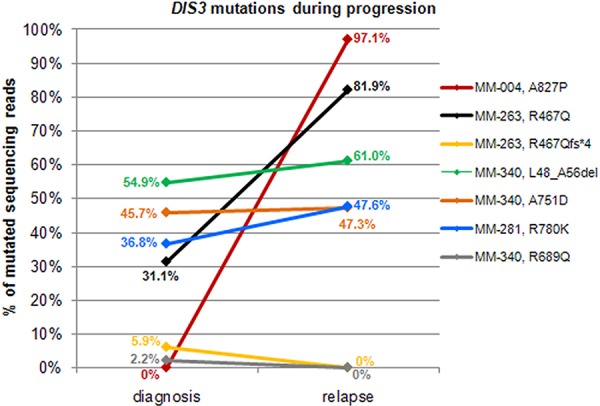
Changes of *DIS3* mutational burden during disease progression For patients found mutated at diagnosis and/or relapse, allele frequencies of variants reported in the legend are plotted at both timepoints.

Variants detected at very low allele frequencies included both missense and frameshift mutations; some of these low-level variants (i.e. D488N, F775L, R820W) were also carried at higher VAFs in other patients. Interestingly, substitutions at residues R780, D487 and D488 (demonstrated or suggested to be particularly detrimental for DIS3 protein function) were never observed at VAFs higher than 50%, neither in the present nor in previously published datasets [6, 7, 13].

### Longitudinal analysis of *DIS3* mutations

To gain further insight into the state of *DIS3* mutations longitudinally, we analyzed 19 patients for whom BM specimens were collected at two different timepoints: in particular, fourteen MM and two pPCL cases collected both at onset and at relapse; two MM patients at onset and at leukemic transformation, and one patient at early and relapsed leukemic phase of MM (Figure 2, Supplementary Table S3). Fifteen out of the 19 cases displayed a wild type *DIS3* status at both timepoints, while one patient (MM-281) carrying the R780K variant showed a quite constant VAF during disease course (36.8% mutated reads at diagnosis, and 47.56% at leukemic transformation). MM-004, wild type at onset, acquired a missense mutation affecting a fully conserved amino acid residue downstream the RNB domain and suggested to be of functional relevance (A827P); this SNV was detected in 97.1% of the sequencing reads at relapse. This patient showed two copies of chromosome 13 both at onset and at relapse, and also the other cytogenetic lesions remained stable during disease progression. A considerable increase in mutation burden was observed in MM-263, with R467Q mutation occurring in 31.1% of reads at onset and 81.9% at relapse (this case also carried a frameshift mutation at the same position with VAF = 5.93% at diagnosis, which disappeared at relapse). Finally, MM-340 carried three variants at onset (L48_A56del, A751D and R689Q at VAF of 54.86%, 45.71% and 2.20%, respectively), two of which (L48_A56del and A751D) remained stable while the third (R689Q) was no longer detectable at relapse. The disappearance of mutations at high allele frequency at onset was never observed in our cohort.

### Expression of *DIS3* variants

**Figure 3:**
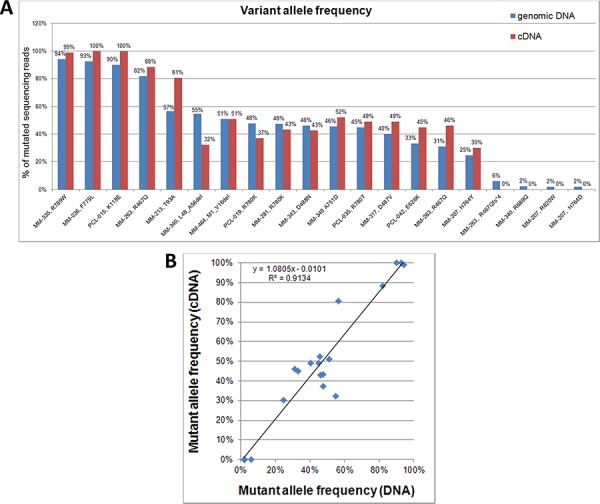
*DIS3* mutations detected on genomic DNA and cDNA **A.** Percentages of variant *DIS3* sequencing reads identified by NGS analyses of genomic DNA and retrotranscribed total RNA. **B.** Correlation between VAFs detected on genomic DNA and cDNA.

To verify whether the *DIS3* mutations here detected were expressed at transcriptional level, we performed the deep sequencing of cDNA samples of the *DIS3*-mutated cases for whom biological material was available. In particular, 20 *DIS3* variants were evaluated at transcriptional level (Figure 3A). Notably, all the variants detected at VAFs higher than 6% on genomic DNA were also identified at RNA level, while none of the four tested low-frequency genomic mutations (VAFs ranging from 1.95% to 5.96%) was detected on cDNA. Overall, as regards expressed variants, mutant allele frequencies detected at gene and transcript level showed a good correlation (Figure 3B). Notably, the aforementioned increase in mutation burden from diagnosis to relapse observed in MM-263 for the variant R467Q was accompanied by a doubling of the amount of expressed mutant allele.

### Transcriptomic profile of *DIS3*-mutated patients

**Figure 4:**
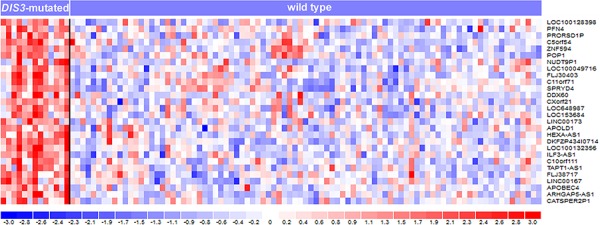
Heatmap of the 28 differentially expressed genes identified at *q*-value = 0 by SAM two-class analysis of 102 MM patients stratified based on the presence of *DIS3* mutations In the heatmap, the color-scale bar represents the relative gene expression changes normalized by the standard deviation, and the color changes in each row represent gene expression level relative to the mean across the samples.

To identify transcriptional gene profiles related to *DIS3* mutations, we investigated by microarray technology a large fraction of MM samples of the present series, including both *DIS3*-mutated and wild type patients. Assuming that alterations present in a very limited number of malignant plasma cells might not appreciably affect gene expression, we chose to set at 20% the lower VAF cut-off to perform supervised analyses. We thus compared 89 wild type and 13 mutated patients for *DIS3*. This analysis revealed that 119 genes (*q*-value < 0.1) (Supplementary Table S4) were differentially expressed between the two groups. Notably, all transcripts were up-regulated in mutated cases (28 of these genes emerged at *q*-value = 0 – Figure 4). Interestingly, 74 were protein-coding genes, while one gene was of unknown type and 44 were non-coding RNAs/pseudogenes. In particular, this latter class of modulated transcripts included nine antisense RNAs (*ARHGAP5-AS1*, *C21orf119*, *DLGAP1-AS1*, *HEXA-AS1*, *ILF3-AS1*, *LOC645212*, *MATN1-AS1*, *TAPT1-AS1*, and *ZNRD1-AS1*), seven pseudogenes (*CATSPER2P1*, *FLJ37201*, *GNRHR2*, *GVINP1*, *HTATSF1P2*, *NUDT9P1*, and *PRORSD1P*), six long intergenic non-protein-coding RNAs (*FLJ30403*, *TP53TG1*, *LINC00167*, *LINC00173*, *LINC00528*, and *LOC100129726*), five small nuclear RNAs (*RNU11*, *RNU12*, *RNU4ATAC*, *RNU5D-1*, and *RNU5F-1*), two microRNAs (*MIR23A* and *MIR320A*), one microRNA-host gene (*MIRLET7BHG*), and a group mainly consisting of uncharacterized *loci* and open reading frames. We then explored whether these transcripts belonged to specific functional subgroups. Remarkably, a significant fraction of them was involved, like *DIS3* itself, in single-stranded RNA binding function (namely, *PAT2L*, *DHX58*, *DDX60* and *KHDRBS2; P* value = 1.01E-04) (Table 3). Beyond this specific molecular function, several other up-regulated genes in *DIS3*-mutated patients are implicated in RNA physiology: in particular, *PAN2*, *POP1*, *RBM45* and the aforementioned *PATL2* and *KHDRBS2* genes are involved in RNA metabolism; *APOBEC3F* and *APOBEC4* encode RNA editing enzymes; whereas *RNU11*, *RNU12* and *RNU4ATAC* are spliceosome subunits. Notably, *DHX58*, *DDX60* and *APOBEC3F* genes, along with *IFIT1*, *IFIT3*, *IFI6*, and *OASL*, are part of interferon-mediated anti-viral response.

**Table 3:** Selected significantly enriched gene ontology terms in the *DIS3* mutation-associated gene signature (*q*-value < 0.05, Benjamini Hochberg correction)

Category	Name	*q*-value	Genes
GO: Molecular Function	single-stranded RNA binding	2.01E-02	*PATL2*, *DHX58*, *DDX60*, *KHDRBS2*
GO: Biological Process	positive regulation of MDA-5 signaling pathway	3.32E-02	*DHX58*, *DDX60*
GO: Biological Process	regulation of MDA-5 signaling pathway	3.32E-02	*DHX58*, *DDX60*
GO: Biological Process	positive regulation of RIG-I signaling pathway	3.32E-02	*DHX58*, *DDX60*
GO: Biological Process	defense response to virus	3.32E-02	*DHX58*, *DDX60*, *IFIT1*, *IFIT3*, *APOBEC3F*, *OASL*
GO: Biological Process	cellular response to type I interferon	3.32E-02	*IFIT1*, *IFIT3*, *OASL*, *IFI6*
GO: Biological Process	type I interferon signaling pathway	3.32E-02	*IFIT1*, *IFIT3*, *OASL*, *IFI6*
GO: Biological Process	MDA-5 signaling pathway	3.32E-02	*DHX58*, *DDX60*
GO: Biological Process	response to type I interferon	3.32E-02	*IFIT1*, *IFIT3*, *OASL*, *IFI6*
GO: Biological Process	regulation of defense response to virus	3.76E-02	*DHX58*, *DDX60*, *IFIT1*, *APOBEC3F*
Pathway (REACTOME)	Interferon alpha/beta signaling	1.01E-04	*IFIT1*, *IFIT3*, *OASL*, *IFI6*

## DISCUSSION

DIS3 belongs to the human exosome complex and is endowed with both exo- and endonucleolytic activities. DIS3 regulates the processing and abundance of all RNA species [18]. Recently, *DIS3* gene has emerged as recurrently mutated in MM patients from initial whole-genome (WGS) and exome (WES) sequencing studies [4, 6, 7, 13, 19]. To the best of our knowledge, this is the first study assessing the *DIS3* mutational status by means of NGS technology in a large cohort of patients including different molecular subtypes and phases (also leukemic stages) of PC dyscrasias and comprehensively evaluating it in the context of other molecular features, and determining the transcriptional profiles and putative altered pathways in mutated MM patients.

*DIS3* mutations were globally identified in 20.1% of patients: specifically, mutation prevalence was 18.5% in newly diagnosed MM patients, 25% in pPCL at onset, and 30% in sPCL. Interestingly, *DIS3* mutation frequency seemed to be slightly higher in extramedullary phases (although this difference did not reach statistical significance, likely due to the relatively small size of pPCL and sPCL series). Notably, a WES study in a fraction of the pPCL patients of the present series identified *DIS3* as one of the 14 statistically significant recurrently affected genes with potential driver role in the disease [20]. As regards the prevalence of *DIS3* mutations identified here in representative MM cases at onset (18.5%), it was higher than the one reported for unselected patients' cohorts in other studies [4, 6, 13]. Most likely, this could be due to the higher depth of coverage of our sequencing analysis, especially compared to that usually obtained in WGS and WES experiments. Compared to the lower frequency (11%) reported by WeiΔbach *et al*. [13], who used our same targeted resequencing approach, this difference could be due to the exclusion in that study of the variants with VAF lower than 8%. We obtained similar results as of Walker *et al*. (18%) [7], who exclusively analyzed *IGH*-translocated patients. Indeed, also in the present series, *DIS3* mutations were confirmed preferentially associated with *IGH* translocations and nonhyperdiploid status.

Of the two functional domains investigated in this study, the RNB domain was slightly more densely affected by mutations. The mutations of the RNB domain initially identified in MM by Chapman *et al*. [4] interfere with DIS3 ribonucleolytic activity [10]. This functional effect could be envisaged also for many of the mutations identified by us, based on the bioinformatics predictions and the pattern of differential expression between *DIS3*-mutated and wild type MM cases emerged from the gene expression profiling of our cohort. *DIS3*-mutated MM samples, in fact, displayed the up-regulation of several transcripts, including genes involved in RNA interactions and many non-coding transcripts. The finding that all the involved transcripts were over-expressed in *DIS3*-mutated patients reflects what reported by several studies analyzing the transcriptional output resulting from the mutation or depletion of exosome subunits in other species, and is consistent with the RNA degradative activities ascribed to the exosome [21–23]. Also the nature of these transcripts, in particular for non-protein-coding RNAs, could be compatible with the accumulation of physiological exosome substrates due to the loss of function of DIS3. In fact, we could notice some similarities between the transcriptional profile characterizing our *DIS3*-mutated MM patients and the genome-wide atlas of *Arabidopsis thaliana* exosome targets presented by Chekanova *et al.,* including hundreds of non-coding RNAs, most of which have not been previously described [21]. Among those targets, Chekanova *et al*. reported upstream non-coding transcripts (UNTs), rRNA precursors, snoRNAs, snRNAs, pseudogenes, pri-miRNAs, and transcripts having neither protein-coding potential nor predicted function [21]. Interestingly, as reported by Tomecki *et al.,* Northern blot analyses in HEK293 cells expressing MM-associated *DIS3* mutations (such as R780K) revealed the accumulation of the majority of analyzed exosome substrates, including 5.8S processing intermediates, tRNAs, RNA polymerase III transcripts and PROMPTs (i.e. PROMoter uPstream Transcripts), these latters generated 0.5 to 2.5 kb upstream of active transcription start sites and rapidly turned over by the RNA exosome [10]. The accumulation of similar nuclear non-coding RNAs (such as cryptic unstable transcripts, CUTs) when MM *DIS3* mutations were expressed in yeast led the same Authors to speculate that the level of ‘transcriptional noise’ is probably much higher in the nucleus of the cells producing DIS3-mutated variants. Concerning the up-regulation of genes involved in interferon-mediated anti-viral response observed in the *DIS3*-mutated MM patients of our cohort, a potential parallel might be suggested between the induction of anti-viral transcriptional response found associated with *DIS3* mutations in our study and the recently reported evidence that SKIV2L, the helicase component of the RNA exosome, is involved in the degradation of endogenous RNAs that might inappropriately trigger the RLRs (RIG-I-like receptors), which are the cytosolic sensors of viral RNAs that initiate host defense, including secretion of type I interferons. Thus, SKIV2L prevents aberrant innate immune responses; remarkably, humans with deficiency in SKIV2L have a type I interferon signature in their peripheral blood [24].

The gene expression patterns observed in our study suggested that identified mutations here are actually expressed, as directly assessed by cDNA sequencing of *DIS3*-mutated patients showing comparable mutation burdens at gene and transcript levels. Overall, these data demonstrate the ultimate significance of the *DIS3* gene mutations, differently from what has been suggested for the majority of the genetic changes detected by genome-wide approaches in MM, which are found in genes that have low or no detectable biological expression [25]. In this regard, it is also worth noting that substitutions at residues R780, D487 and D488 (demonstrated or supposed to be particularly detrimental for protein function) were never observed at genomic VAFs above 50%, neither in the present nor in previously published datasets [6, 7, 13], suggesting that the exclusive presence of such *DIS3* mutant forms in the absence of a *DIS3* wild type protein might have deleterious cellular effects. In general, the lack of a *DIS3* wild type allele in mutated cases was uniquely observed in some 13q14-deleted MM patients carrying other types of variants.

Furthermore, looking at *DIS3* mutations in longitudinally analyzed patients (i.e. sampled at diagnosis and relapse), we observed a similar scenario to that described by Bolli *et al*. [3] for other known driver myeloma genes, i.e. the occurrence of clonal variants at both timepoints or the acquisition/clonal expansion of variants at the later timepoint, consistently with an expected positive selection for the subclones harboring them. Of note, the disappearance at relapse of variants found at diagnosis at low allele frequency was only observed in patients carrying additional stable/clonally expanding mutations. The limited sample size and the different treatment regimens to which the patients were subjected do not allow making assumptions on the possible impact of specific therapies on the chances of survival of *DIS3*-mutated subclones.

The clinical implications of *DIS3* mutations in MM remain to be elucidated. Recently, WeiΔbach *et al*. [13] reported that the occurrence of *DIS3* mutations in minor subclones was significantly associated with a weaker chemotherapy response as compared to the *DIS3* mutations in major subclones, while globally there was only a slight trend towards shorter median overall survival for *DIS3*-mutated MM patients as compared to *DIS3* wild type ones. In our series, we had the possibility to evaluate the prognostic impact of *DIS3* mutations in the group of pPCLs included in a prospective multicenter clinical trial [26] for whom a 2.8-month follow-up was available, observing no significant association with survival or response rate (data not shown).

In conclusion, our data confirm and extend the evidence that *DIS3* mutations are recurrent in MM, suggesting an even greater involvement of *DIS3* alterations in more advanced stages of PC dyscrasias, and highlighting some instances of increase of *DIS3* mutation burden during disease progression. Although its role in the pathogenesis of the disease remains to be further elucidated, *DIS3* is considered a potential tumor suppressor in MM. This suggestion is based on the loss of enzymatic activity caused by the MM-associated *DIS3* mutations that have been functionally characterized [10]; the loss of heterozigosity/disruption (as also elucidated in our study) often involving *DIS3* due to the combination of gene mutations and chromosome 13 deletion; and the increased translation of critical oncogenes observed as one of the biological outcomes of DIS3 inactivation [14]. Importantly, the transcriptional phenotype observed here in *DIS3*-mutated MM patients could be consistent with an impaired function of the RNA exosome complex, thus further supporting the pathological role of *DIS3* alterations in the disease.

## MATERIALS AND METHODS

### Patients and cell lines

After the patients had given their informed consent in accordance with institutional guidelines (clearance from Ethic Committee, Fondazione Ospedale Maggiore di Milano, Italy), pathological BM specimens were obtained during standard diagnostic procedures from 130 newly diagnosed MM patients, 8 pPCL cases at onset, and 10 sPCLs, admitted from July 2001 to April 2014. Additional 16 pPCL patients were included in a multicenter clinical trial (RV-PCL-PI-350, EudraCT N°2008-003246-28) [26]. Seventy-seven patients were males; median age was 66 years (range: 42–85). The diagnosis of MM and PCL was made according to the previously described criteria [2, 27]. Ninety-nine patients had an immunoglobulin (Ig) G protein monoclonal component; 34 IgA; one IgG/IgA; and one IgM protein; 99 cases had the light chain κ; 62 λ; and two λ+κ. Twenty-five MM cases were in stage IA, 57 in IIA/B and 48 in IIIA/B, according to Durie and Salmon criteria [28]. Many of the patients included in this study have been described in our previous reports [29, 30].

HMCLs investigated in this study were the following: NCI-H929, OPM2, JJN3, KMS-12, KMS-28, KMS-34, KMS-18, KMS-11, KMS-26, AMO1, RPMI 8226, DELTA-47, SK-MM-1, UTMC-2, MM.1S, U266, CMA-03 and CMA-03/06, LP-1, KMS-27. With the exception of DELTA-47, UTMC-2 and MM.1S, all the others have been previously described and reported by us [31, 32].

### Sample preparation and molecular analyses

The BM specimens were collected from patients at the time of diagnosis; 19 cases were re-sampled at relapse/MM leukemic transformation (median time interval between the two samplings: 30 months). PCs were purified (≥ 90% in all cases) using CD138 immunomagnetic microbeads as previously described [33, 34]. All cases were characterized by fluorescence *in situ* hybridization (FISH) for the main genomic aberrations (namely, *IGH* translocations, hyperdiploidy, del(13q), del(17p), del(1p) and 1q gain) [16] (Supplementary Table S5), and by NGS for the mutational status of *BRAF*, *NRAS* and *KRAS* genes [8].

### Mutation analyses

NGS of *DIS3* exons 1–4 (PIN domain) and exons 10–18 (RNB domain) (RefSeq NM_014953.4, representing the longer transcript encoding the longest protein isoform) was performed on genomic DNA using the Genome Sequencer Junior instrument (Roche-454 Life Sciences, Penzberg, Germany), as previously described [35]. Further details on primer sequences and sequencing protocol are available in the Supplementary Materials and Methods and Supplementary Table S6 and S7. The obtained sequencing reads were mapped to the *DIS3* human reference sequence (RefSeq NC_000013.10) and analyzed by the Amplicon Variant Analyzer (AVA) software version 3.0 (454 Life Sciences) to establish the variant allele frequency.

The presence of each obtained non-synonymous variant was verified in an independent PCR product by conventional sequencing whenever the sensitivity of the Sanger method was consistent with the VAF. NGS analysis was repeated in case of mutations detected in less than 10% of sequencing reads. To exclude germline variants, we sequenced the matched normal DNA, when available, or consulted the NCBI Human dbSNP Database (Build 142, http://www.ncbi.nlm.nih.gov/snp). The occurrence of *DIS3* variants was also verified at transcriptional level (see Supplementary Materials and Methods).

Additional information about the functional relevance of the detected SNVs was obtained using five bioinformatics tools which calculate possible impact of amino acid substitutions on the structure and function of human proteins using straightforward physical and evolutionary comparative considerations. The functional predictors used were Polyphen2 (http://genetics.bwh.harvard.edu/pph2/), Mutation Taster (http://www.mutationtaster.org/), Mutation Assessor (http://mutationassessor.org/), SIFT (http://sift.jcvi.org/) and LRT (http://www.genetics.wustl.edu/jflab/lrt_query.html).

### Gene expression profiling

For gene expression profiling (GEP), samples were profiled on the GeneChip Human Gene 1.0 ST array (Affymetrix, Santa Clara, CA, USA), as previously described [30]. Supervised analyses were performed using Significant Analysis of Microarrays software (SAM version 4.00; Excel front-end publicly available at http://www-stat.stanford.edu/tibs/SAM/index.html) [36]. The functional annotation analysis of selected gene lists was performed by means of DAVID 6.7 tool (http://david.abcc.ncifcrf.gov/) and the ToppFun option of ToppGene Suite (https://toppgene.cchmc.org/), using the default parameters. Details on GEP data generation and analysis are included in the Supplementary Materials and Methods. The GEP data have been deposited in the NCBI Gene Expression Omnibus database (GEO; http//www.ncbi.nlm.nih.gov/geo; accession No. GSE66293).

### Statistical analyses

All contingency analyses were performed by two-sided Fisher's exact test (*P* value < 0.05).

## SUPPLEMENTARY MATERIALS AND METHODS FIGURES AND TABLES


