# Supplementary material for: A compendium of DIS3 mutations and associated transcriptional signatures in plasma cell dyscrasias
Source: Oncotarget. 2015 Jul 20;6(28):26129–41. doi: 10.18632/oncotarget.4674 (PMC4694891; doi:10.18632/oncotarget.4674)
Supplement: Supplementary file 1 [file oncotarget-06-26129-s001.pdf]

SUPPLEMENTARY MATERIAL

A compendium of *DIS3* mutations and associated transcriptional signatures in plasma cell dyscrasias

Supplementary Materials and Methods 2
Supplementary Figures 3
Supplementary Tables 5

SUPPLEMENTARY MATERIALS AND METHODS

Mutation analyses

Genomic DNA was extracted using Wizard genomic purification DNA kit (Promega Corporation, Madison, WI, USA) according to manufacturer's instructions, spectrophotometrically quantified and amplified using FastStart High Fidelity Polymerase (Roche) and fusion primers (Roche) containing M13 adapter sequences and the sequence-specific primers (Supplementary Table S6) spanning *DIS3* exons 1–4 for the PIN domain and exons 10–18 for the RNB domain (RefSeq NM_014953.4, representing the longer transcript encoding the longest protein isoform).

Amplicon library A and B sequencing adapters and multiplex identifier (MID) tags were then added to both tails of amplicons by a second amplification step. PCR conditions were as follows: in the first amplification step, denaturation step at 94°C for 5 min. followed by 25 cycles at 94°C (30 sec. per cycle), annealing step at 53°C (for exon 1), 57°C (for exons 10, 11 and 17), or 58°C (for the remaining exons) (30 sec. per cycle), and extension at 72°C (45 sec. per cycle), followed by a final extension at 72°C for 7 min.; in the second amplification step, denaturation step at 94°C for 5 min. followed by 25 cycles at 94°C (20 sec. per cycle), annealing step at 55°C (20 sec. per cycle), and extension at 72°C (45 sec. per cycle), followed by a final extension at 72°C for 10 min.. PCR products were visualized on agarose gel, purified using AMPure XP DNA-binding paramagnetic beads (Agencourt Bioscience Corp., Beckman Coulter S.p.A, Milan, Italy), and quantified using picogreen dye (Life Technologies, Carlsbad, California) and the Victor X2 (Perkin Elmer, Waltham, Massachusetts) fluorometer. Samples were then pooled together at equimolar ratios to prepare for Roche/454 pyrosequencing. The obtained amplicon library was added to the emulsion PCR at a ratio of 0.8 molecules per bead and subjected to deep sequencing on the Genome Sequencer Junior instrument (Roche-454 Life Sciences). The obtained sequencing reads were mapped to the *DIS3* human reference sequence (RefSeq NC_000013.10) and analyzed by the Amplicon Variant Analyzer (AVA) software version 3.0 (Roche-454 Life Sciences) to establish the mutant allele frequency.

To verify the occurrence of *DIS3* variants at transcriptional level, total RNA of mutated samples was converted to cDNA using M-MLV reverse transcriptase (Invitrogen Life Technologies, Carlsbad, California) and random hexamers, and subjected to deep sequencing of the exon harboring the variant/s detected on genomic DNA. Sequence-specific exonic PCR primers were designed in the Primer 3 program (http://frodo.wi.mit.edu/primer3/) and are reported in Supplementary Table S7.

Gene expression profiling and data analysis

For gene expression profiling (GEP), samples were profiled on the GeneChip Human Gene 1.0 ST array (Affymetrix, Santa Clara, CA, USA), as previously described [1]. Briefly, the raw intensity expression values were processed by Robust Multi-array Average procedure (RMA) [2], with the re-annotated Chip Definition Files from BrainArray libraries version 18.0.0 [3], available at http://brainarray.mbni.med.umich.edu. Supervised analyses were performed using Significant Analysis of Microarrays software (SAM version 4.00; Excel front-end publicly available at http://www-stat.stanford.edu/tibs/SAM/index.html) [4] as previously described [5]. The cutoff point for statistical significance (q-value < 10%) was determined by tuning the Δ parameter on the false discovery rate (FDR) and controlling the q-value of the selected probes. Differentially expressed genes were also evaluated at higher stringency level (median FDR 0%). The functional annotation analysis on the selected lists was performed by means of DAVID 6.7 tool (http://david.abcc.ncifcrf.gov/) and the ToppFun option of ToppGene Suite (https://toppgene.cchmc.org/), using the default parameters.

REFERENCES

1. Todoerti K, Agnelli L, Fabris S, Lionetti M, Tuana G, Mosca L, Lombardi L, Grieco V, Bianchino G, D'Auria F, Statuto T, Mazzoccoli C, De Luca L, et al. Transcriptional characterization of a prospective series of primary plasma cell leukemia revealed signatures associated with tumor progression and poorer outcome. Clin Cancer Res. 2013; 19:3247–3258.
2. Irizarry RA, Hobbs B, Collin F, Beazer-Barclay YD, Antonellis KJ, Scherf U, Speed TP. Exploration, normalization, and summaries of high density oligonucleotide array probe level data. Biostatistics. 2003; 4:249–264.
3. Dai MH, Wang PL, Boyd AD, Kostov G, Athey B, Jones EG, Bunney WE, Myers RM, Speed TP, Akil H, Watson SJ, Meng F. Evolving gene/transcript definitions significantly alter the interpretation of GeneChip data. Nucleic Acids Res. 2005; 33:e175.
4. Tusher VG, Tibshirani R, Chu G. Significance analysis of microarrays applied to the ionizing radiation response. Proc Natl Acad Sci U S A. 2001; 98:5116–5121.
5. Agnelli L, Fabris S, Bicciato S, Basso D, Baldini L, Morabito F, Verdelli D, Todoerti K, Lambertenghi-Deliliers G, Lombardi L, Neri A. Upregulation of translational machinery and distinct genetic subgroups characterise hyperdiploidy in multiple myeloma. Br J Haematol. 2007; 136:565–573.

SUPPLEMENTARY FIGURES AND TABLES

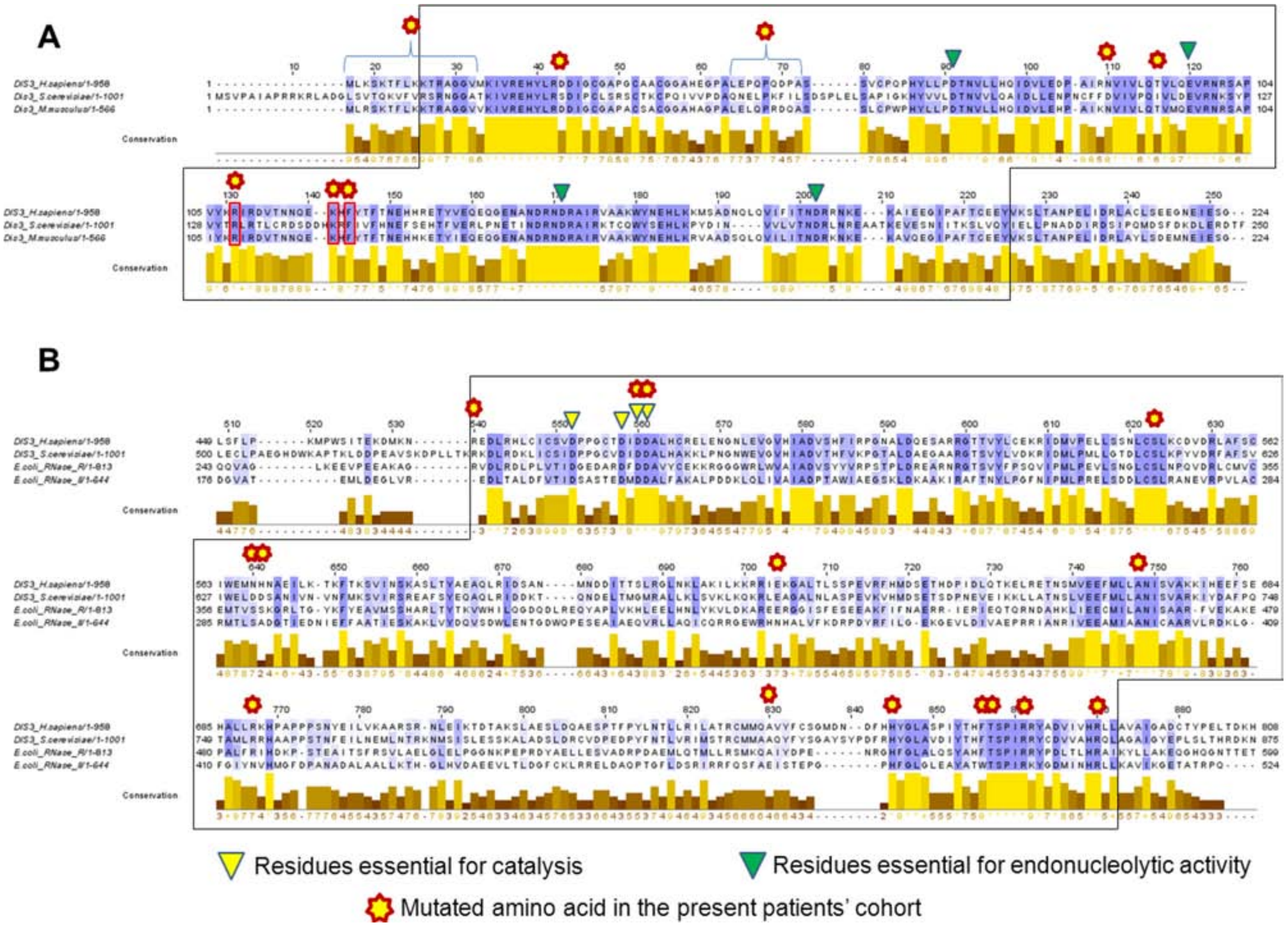

Supplementary Figure S1: Detailed amino acid sequence alignment of DIS3 proteins of various species. In particular, human, yeast and murine DIS3 PIN domains were compared **(A),** and human and yeast RNB domains were aligned with *E.coli* RNAses II and R **(B).** Each domain is bordered by a black frame. Stars indicate genic positions targeted by missense mutations or in-frame deletions in the present primary patients' series. Green triangles indicate acidic residues predicted to coordinate a divalent cation and essential for endonucleolytic activity. Yellow triangles indicate residues involved in binding the magnesium ion at the active site. Sequence alignment was performed by Jalview software v2.7.

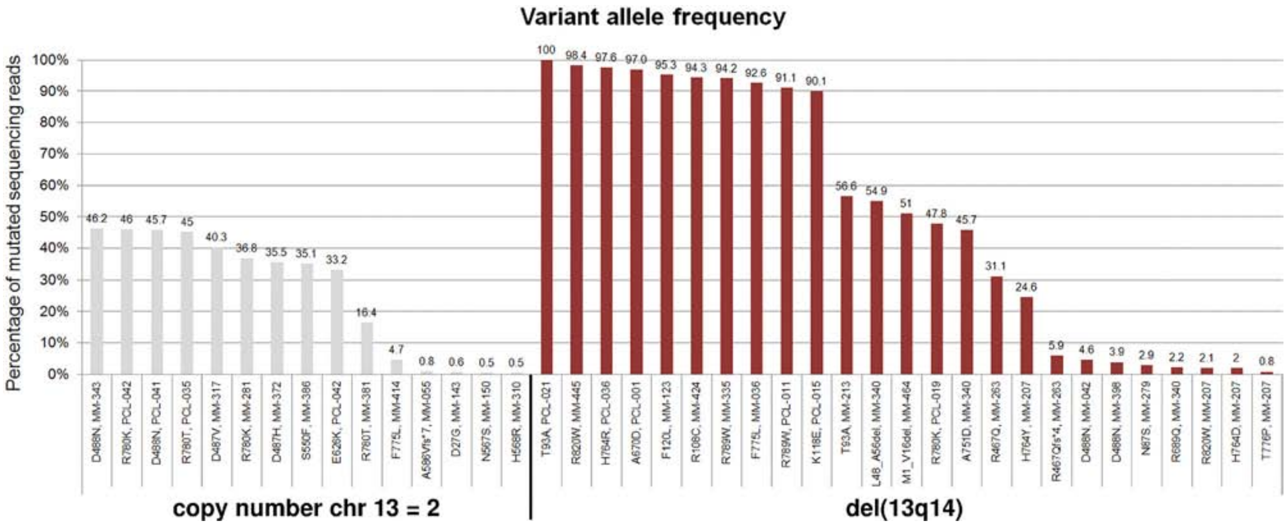

Supplementary Figure S2: Percentage of variant *DIS3* sequencing reads on total sequencing reads for the non-synonymous somatic mutations identified by NGS analysis in primary patients. Horizontal axis: sample id and carried amino acid variant are reported; patients are ordered according to copy number of chromosome 13 and decreasing mutation load.

Supplementary Table S1. Prediction of the functional relevance of the 26 missense *DIS3* mutations found in the present primary patients' cohort

AA change	Polyphen2	Mutation Taster	Mutation Assessor [FI score]	SIFT PREDICTION (SIFT score)	LRT (Likelihood Ratio test)
**D27G**	PROBABLY DAMAGING	DISEASE CAUSING	PREDICTED FUNCTIONAL (MEDIUM) [3.165]	DAMAGING (0.01)	DELETERIOUS
**N87S**	PROBABLY DAMAGING	DISEASE CAUSING	PREDICTED FUNCTIONAL (MEDIUM) [2.98]	DAMAGING (0.03)	DELETERIOUS
**T93A**	POSSIBLY DAMAGING	DISEASE CAUSING	PREDICTED FUNCTIONAL (MEDIUM) [3.34]	DAMAGING (0)	DELETERIOUS
**R108C**	PROBABLY DAMAGING	DISEASE CAUSING	PREDICTED FUNCTIONAL (MEDIUM) [3.45]	DAMAGING (0)	NEUTRAL
**K118E**	POSSIBLY DAMAGING	DISEASE CAUSING	PREDICTED FUNCTIONAL (MEDIUM) [3.24]	DAMAGING (0.01)	NEUTRAL
**F120L**	PROBABLY DAMAGING	DISEASE CAUSING	PREDICTED FUNCTIONAL (MEDIUM) [3.065]	DAMAGING (0.05)	DELETERIOUS
**R467Q**	PROBABLY DAMAGING	DISEASE CAUSING	PREDICTED FUNCTIONAL (HIGH) [4.675]	DAMAGING (0)	DELETERIOUS
**D487H**	PROBABLY DAMAGING	DISEASE CAUSING	PREDICTED FUNCTIONAL (HIGH) [4.74]	DAMAGING (0)	DELETERIOUS
**D487V**	PROBABLY DAMAGING	DISEASE CAUSING	PREDICTED FUNCTIONAL (HIGH) [4.74]	DAMAGING (0)	DELETERIOUS

(*Continued*)

AA change	Polyphen2	Mutation Taster	Mutation Assessor [FI score]	SIFT PREDICTION (SIFT score)	LRT (Likelihood Ratio test)
**D488N**	PROBABLY DAMAGING	DISEASE CAUSING	PREDICTED FUNCTIONAL (HIGH) [4.74]	DAMAGING (0)	DELETERIOUS
**S550F**	PROBABLY DAMAGING	DISEASE CAUSING	PREDICTED FUNCTIONAL (HIGH) [4.775]	DAMAGING (0)	DELETERIOUS
**N567S**	BENIGN	DISEASE CAUSING	PREDICTED NON-FUNCTIONAL (NEUTRAL) [-0.345]	TOLERATED (0.19)	DELETERIOUS
**H568R**	BENIGN	DISEASE CAUSING	PREDICTED NON-FUNCTIONAL (NEUTRAL) [-0.455]	TOLERATED (0.26)	NEUTRAL
**E626K**	BENIGN	DISEASE CAUSING	PREDICTED NON-FUNCTIONAL (LOW) [0.835]	TOLERATED (0.63)	DELETERIOUS
**A670D**	PROBABLY DAMAGING	DISEASE CAUSING	PREDICTED FUNCTIONAL (HIGH) [4.74]	DAMAGING (0)	DELETERIOUS
**R689Q**	PROBABLY DAMAGING	DISEASE CAUSING	PREDICTED FUNCTIONAL (HIGH) [4.77]	DAMAGING (0)	DELETERIOUS
**A751D**	PROBABLY DAMAGING	DISEASE CAUSING	PREDICTED FUNCTIONAL (HIGH) [4.77]	DAMAGING (0)	DELETERIOUS
**H764D**	PROBABLY DAMAGING	DISEASE CAUSING	PREDICTED FUNCTIONAL (HIGH) [4.77]	DAMAGING (0)	DELETERIOUS

(*Continued*)

AA change	Polyphen2	Mutation Taster	Mutation Assessor [FI score]	SIFT PREDICTION (SIFT score)	LRT (Likelihood Ratio test)
**H764R**	PROBABLY DAMAGING	DISEASE CAUSING	PREDICTED FUNCTIONAL (HIGH) [4.77]	DAMAGING (0)	DELETERIOUS
**H764Y**	PROBABLY DAMAGING	DISEASE CAUSING	PREDICTED FUNCTIONAL (HIGH) [4.77]	DAMAGING (0)	DELETERIOUS
**F775L**	PROBABLY DAMAGING	DISEASE CAUSING	PREDICTED FUNCTIONAL (HIGH) [4.77]	DAMAGING (0)	DELETERIOUS
**T776P**	PROBABLY DAMAGING	DISEASE CAUSING	PREDICTED FUNCTIONAL (HIGH) [4.77]	DAMAGING (0)	DELETERIOUS
**R780K**	PROBABLY DAMAGING	DISEASE CAUSING	PREDICTED FUNCTIONAL (HIGH) [4.77]	DAMAGING (0)	DELETERIOUS
**R780T**	PROBABLY DAMAGING	DISEASE CAUSING	PREDICTED FUNCTIONAL (HIGH) [4.77]	DAMAGING (0)	DELETERIOUS
**R789W**	POSSIBLY DAMAGING	DISEASE CAUSING	PREDICTED FUNCTIONAL (HIGH) [4.77]	DAMAGING (0)	DELETERIOUS
**R820W**	PROBABLY DAMAGING	DISEASE CAUSING	PREDICTED FUNCTIONAL (MEDIUM) [3.275]	DAMAGING (0)	DELETERIOUS

Supplementary Table S2. Clinical and molecular characteristics of the 33 MM/PCL patients carrying *DIS3* non-synonymous mutations or indels

Sample name	Disease stage[†]	Variant*	Mutated reads (%)	AA change	del (13)	del (17p)	1q gain	1p loss	t(4;14)	t(11;14)	t(14;16)	t(14;20)	t(6;14)	HD[‡]
**MM-036**	MM	73336078A>C	92.57%	F775L	+	–	+	nd	–	–	–	–	–	–
**MM-042**	MM	73346338C>T	4.57%	D488N	+	–	+	–	+	–	–	–	–	+
**MM-055**	MM	73343049_ 73343050insA	0.83%	A586Vfs*7	–	–	–	nd	–	+	–	–	–	–
**MM-123**	MM	73355010G>C	95.30%	F120L	+	–	+	–	+	–	–	–	–	–
**MM-143**	MM	73355891T>C	0.62%	D27G	–	–	+	–	–	–	–	–	–	+
**MM-150**	MM	73345097T>C	0.51%	N567S	–	–	+	–	–	–	–	–	–	+
**MM-207**	MM	73336113G>A<br>73335837G>A<br>73336113G>C<br>73336077T>G	24.58%<br>2.05%<br>1.95%<br>0.79%	H764Y<br>R820W<br>H764D<br>T776P	+	–	+	–	–	–	–	–	+	–
**MM-213**	MM	73355093T>C	56.57%	T93A	+	–	–	–	–	+	–	–	–	–
**MM-263**	MM	73346400C>T<br>73346400del	31.14%<br>5.93%	R467Q<br>R467Qfs*4	+	–	+	–	+	–	–	–	–	–
**MM-279**	MM	73355110T>C	2.93%	N87S	+	–	+	–	–	–	–	nd	nd	nd
**MM-281**	MM	73336064C>T	36.80%	R780K	–	–	–	–	–	–	–	–	–	–
**MM-310**	MM	73345094T>C	0.50%	H568R	–	–	–	–	–	+	–	–	–	–
**MM-335**	MM	73335930G>A	94.20%	R789W	+	–	–	–	–	–	+	–	–	–

(*Continued*)

Sample name	Disease stage†	Variant*	Mutated reads (%)	AA change	del (13)	del (17p)	1q gain	1p loss	t(4;14)	t(11;14)	t(14;16)	t(14;20)	t(6;14)	HD‡
**MM-340**	MM	73355804_73355830del<br>73336151G>T<br>73337650C>T	54.86%<br>45.71%<br>2.2%	L48_A56del<br>A751D<br>R689Q	+	–	–	–	–	+	–	–	–	–
**MM-343**	MM	73346338C>T	46.18%	D488N	–	–	–	–	–	+	–	–	–	–
**MM-372**	MM	73346341C>G	35.48%	D487H	–	–	+	–	–	+	–	–	–	–
**MM-381**	MM	73336064C>G	16.38%	R780T	–	–	–	–	–	–	–	nd	nd	nd
**MM-386**	MM	73345240G>A	35.11%	S550F	–	–	nd	nd	–	nd	nd	nd	nd	nd
**MM-398**	MM	73346338C>T	3.85%	D488N	+	–	+	–	–	+	–	–	–	–
**MM-414**	MM	73336078A>C	4.71%	F775L	–	–	–	–	–	+	–	–	–	–
**MM-424**	MM	73355048G>A	94.33%	R108C	+	–	–	+	–	–	–	–	–	+
**MM-445**	MM	73335837G>A	98.36%	R820W	+	–	+	–	–	+	–	–	nd	–
**MM-464**	MM	73355968C>T	50.99%	M1_V16del	+	–	+	–	–	–	–	–	–	–
**MM-317**	MM	73346340T>A	40.3%	D487V	–	–	+	–	–	+	–	–	–	–
**PCL-001**	pPCL	73337707G>T	97%	A670D	+	–	nd	nd	–	–	–	+	–	nd
**PCL-015**	pPCL	73355018T>C	90.05%	K118E	+	–	+	–	–	–	+	–	–	–
**PCL-019**	pPCL	73336064C>T	47.75%	R780K	+	+	+	+	–	–	+	–	–	–
**PCL-021**	pPCL	73355093T>C	100%	T93A	+	–	+	–	+	–	–	–	–	–

(*Continued*)

Sample name	Disease stage†	Variant*	Mutated reads (%)	AA change	del (13)	del (17p)	1q gain	1p loss	t(4;14)	t(11;14)	t(14;16)	t(14;20)	t(6;14)	HD‡
**PCL-035**	pPCL	73336064C>G	45.04%	R780T	–	–	–	–	–	+	–	–	–	–
**PCL-036**	pPCL	73336112T>C	97.59%	H764R	+	–	–	–	–	+	–	–	–	–
**PCL-011**	sPCL	73335930G>A	91.1%	R789W	+	+	+	–	–	–	+	–	–	–
**PCL-041**	sPCL	73346338C>T	45.68%	D488N	–	–	nd	nd	–	nd	nd	nd	nd	nd
**PCL-042**	sPCL	73336064C>T<br>73342930C>T	45.95%<br>33.15%	R780K<br>E626K	–	–	–	–	–	+	–	–	–	–

† MM: multiple myeloma; pPCL: primary plasma cell leukemia; sPCL: secondary plasma cell leukemia.
* Genomic positions based on hg19. ‡HD: presence of the hyperdiploid status on the basis of FISH evaluation criteria

Supplementary Table S3. Mutational status of *DIS3* gene in 19 longitudinally analyzed patients

Sample name	Disease stage	*DIS3* status
**MM-004**	MM onset	wild type
	MM relapse	A827P, 97.1%
**MM-146**	MM onset	wild type
	MM relapse	wild type
**MM-151**	MM onset	wild type
	MM relapse	wild type
**MM-200**	MM onset	wild type
	MM relapse	wild type
**MM-239**	MM onset	wild type
	MM relapse	wild type
**MM-263**	MM onset	R467Q, 31.1%; R467Qfs*4, 5.9%
	MM relapse	R467Q, 81.9%
**MM-271**	MM onset	wild type
	MM relapse	wild type
**MM-280**	MM onset	wild type
	MM relapse	wild type
**MM-282**	MM onset	wild type
	MM relapse	wild type
**MM-286**	MM onset	wild type
	MM relapse	wild type
**MM-327**	MM onset	wild type
	MM relapse	wild type
**MM-334**	MM onset	wild type
	MM relapse	wild type
**MM-340**	MM onset	L48_A56del, 54.9%; A751D, 45.7%; R689Q, 2.2%
	MM relapse	L48_A56del, 61%; A751D, 42.3%
**MM-429**	MM onset	wild type
	MM relapse	wild type
**MM-295**	MM onset	wild type
	MM leukemic transformation	wild type
**MM-281**	MM onset	R780K, 36.8%
	MM leukemic transformation	R780K, 47.6%
**PCL-026**	pPCL onset	wild type
	pPCL relapse	wild type

(*Continued*)

Sample name	Disease stage	*DIS3* status
**PCL-038**	pPCL onset	wild type
	pPCL relapse	wild type
**MM-442**	MM leukemic transformation	wild type
	sPCL relapse	wild type

Supplementary Table S4. Modulated genes between *DIS3*- wild type and mutated patients

Gene	SAM score
LOC100132356	−7.27998
DKFZP434I0714	−7.24885
ILF3-AS1	−5.8891
FLJ30403	−5.73335
ARHGAP5-AS1	−5.63017
HEXA-AS1	−5.52727
C5orf54	−5.41888
C11orf71	−5.0731
ZNF594	−5.01186
FLJ38717	−4.90146
LOC100049716	−4.82276
CXorf21	−4.79455
LOC648987	−4.75759
TAPT1-AS1	−4.65361
C10orf111	−4.55991
APOBEC4	−4.54461
PRORSD1P	−4.35225
LINC00167	−4.34833
DDX60	−4.29767
CATSPER2P1	−4.21904
SPRYD4	−4.19583
POP1	−4.13861
PFN4	−4.08844
LINC00173	−3.94784
LOC153684	−3.93605
APOLD1	−3.92105
LOC100128398	−3.87278
NUDT9P1	−3.83567
C1orf220	−3.6607
RNU12	−3.64519
HTATSF1P2	−3.62834
DHX58	−3.61677
RNU11	−3.61058
CENPM	−3.58134
STPG2	−3.54494
FAM218A	−3.50969

(*Continued*)

Gene	SAM score
FREM3	−3.49656
FIGN	−3.47013
IFIT3	−3.46587
ZNF268	−3.46146
NS3BP	−3.40966
ANXA2R	−3.36509
LDOC1L	−3.3559
C21orf119	−3.34986
C11orf65	−3.33332
FLJ37201	−3.32968
LOC148696	−3.32702
DPM3	−3.30907
SLC4A5	−3.2872
LOC100506571	−3.28623
FLJ38576	−3.26721
PLA2G6	−3.24009
ZNRD1-AS1	−3.23349
PCDHGA1	−3.22484
PATL2	−3.21516
C9orf43	−3.21034
RNU5D-1	−3.20768
IL22RA1	−3.20672
C16orf54	−3.20079
SCIN	−3.1948
C5orf56	−3.18962
LINC00528	−3.17856
RBM45	−3.1685
GVINP1	−3.16364
KHDRBS2	−3.14396
MTG1	−3.1248
FAM154B	−3.12346
ST3GAL1	−3.12014
LOC100128288	−3.10885
C7orf13	−3.10235
TTC30B	−3.10072
CXXC4	−3.09534

(*Continued*)

Gene	SAM score
IFIT1	−3.08992
MIR320A	−3.08972
RNU4ATAC	−3.08157
PAPSS2	−3.07687
HSD17B7	−3.07454
MATN1-AS1	−3.0743
LRRC23	−3.05173
ZNF141	−3.04218
MAP2	−3.02161
MED31	−3.01969
SFR1	−3.01663
APOBEC3F	−3.01397
GDF9	−3.01336
MPZ	−2.99457
LOC645212	−2.98619
CDNF	−2.98353
C1orf74	−2.97908
TP53TG1	−2.97753
RASL11B	−2.97566
LOC100129726	−2.97513
GNRHR2	−2.97302
GPR135	−2.96435
OASL	−2.96341
CCDC87	−2.94704
HCG27	−2.93779
RNU5F-1	−2.92498
FAM227B	−2.91766
MIR23A	−2.91476
MIRLET7BHG	−2.91089
RIBC2	−2.8987
ZBTB26	−2.89665
PAN2	−2.89352
FZD7	−2.87535
CCDC85C	−2.87272
DLGAP1-AS1	−2.86802
HIST4H4	−2.85887

(*Continued*)

Gene	SAM score
KANSL1L	−2.85357
IFI6	−2.84554
EID2B	−2.83955
CCDC53	−2.83361
DDX60L	−2.82877
TTC23	−2.81725
ZNF70	−2.81337
COQ2	−2.81171
ODF3B	−2.80322
AGBL2	−2.79022
TMEM234	−2.77988

Supplementary Table S5. Clinical details of the 164 patients analyzed by NGS

Sample	*Sex*	*Age*	*Disease*	*Stage*[Δ]	*Phase*[‡]	*PP*[†]	*t(4;14)*	*t(11;14)*	*t(14;16)*	*t(14;20)*	*del(13)**	*del(17p)**	*1q gain**	*1p loss**	*HD°*
**MM-004**	F	58	MM	IA	D	Gκ	−	−	+	−	−	na	na	na	na
**MM-015**	M	71	MM	IIA	D	Gκ	−	+	−	−	−	−	−	−	−
**MM-016**	M	66	MM	IIIB	D	Gκ	−	−	−	−	−	−	+	−	+
**MM-026**	F	72	MM	IIIB	D	κ	−	+	−	−	−	−	−	na	na
**MM-027**	M	60	MM	IA	D	Gκ	−	−	−	−	+	−	na	na	na
**MM-030**	M	69	MM	IIIA	D	Gλ	−	−	−	−	−	−	−	+	+
**MM-031**	M	58	MM	IIIA	D	Aκ	−	+	−	−	−	−	+	+	−
**MM-034**	M	71	MM	IA	D	Gκ	−	−	−	−	−	−	−	−	+
**MM-036**	M	65	MM	IIA	D	Gκ	−	−	−	−	+	−	+	na	−
**MM-037**	F	50	MM	IIA	D	Gκ+Aκ	−	+	−	−	+	−	−	−	−
**MM-038**	F	67	MM	IIA	D	κ	−	−	−	−	+	−	−	−	+
**MM-039**	M	50	MM	IIA	D	Gλ	−	−	−	−	−	−	−	na	+
**MM-042**	M	54	MM	IIIA	D	Aλ	+	−	−	−	+	−	+	−	+
**MM-043**	F	73	MM	IA	D	Gκ+Gλ	−	−	−	−	−	−	+	−	−
**MM-049**	M	62	MM	IIIB	D	κ	−	−	−	−	−	−	−	−	+
**MM-055**	F	69	MM	IIIA	D	Gκ	−	+	−	−	−	−	−	na	−
**MM-066**	F	77	MM	IIIA	D	Aκ	+	−	−	−	−	−	−	na	−
**MM-069**	M	63	MM	IIA	D	Gκ	−	−	−	+	+	−	+	na	−
**MM-078**	F	59	MM	IIIA	D	κ	−	−	−	−	+	−	−	na	+
**MM-079**	F	74	MM	IIA	D	Gκ+Gλ	−	−	−	−	−	−	−	−	+
**MM-087**	F	84	MM	IIIA	D	Gλ	+	−	−	−	+	−	+	na	−
**MM-115**	F	53	MM	IIIA	D	Gλ	−	+	−	−	+	−	na	na	−
**MM-123**	M	55	MM	IIIA	D	Gκ	+	−	−	−	+	−	+	−	−
**MM-131**	M	73	MM	IA	D	Gκ	−	−	−	−	−	−	−	−	−
**MM-143**	M	61	MM	IIA	D	Gκ	−	−	−	−	−	−	+	−	+
**MM-146**	M	68	MM	IIA	D	Gλ	−	−	−	−	−	−	+	na	+

(*Continued*)

Sample	Sex	Age	Disease	Stage[Δ]	Phase[‡]	PP[†]	t(4;14)	t(11;14)	t(14;16)	t(14;20)	del(13)*	del(17p)*	1q gain*	1p loss*	HD°
**MM-148**	F	55	MM	IA	D	Aκ	−	−	−	−	−	+	+	na	+
**MM-149**	F	52	MM	IA	D	Gλ	−	−	−	−	−	−	−	−	+
**MM-150**	F	68	MM	IIA	D	Gλ	−	−	−	−	−	−	+	−	+
**MM-151**	F	71	MM	IA	D	Gλ	−	−	−	−	−	−	−	−	+
**MM-152**	M	66	MM	IA	D	Gκ	−	−	−	−	−	−	−	na	+
**MM-154**	F	71	MM	IIA	D	Gκ	−	−	+	−	+	−	+	−	−
**MM-159**	M	56	MM	IIA	D	κ	−	+	−	−	+	−	−	−	−
**MM-174**	M	85	MM	IIA	D	Aκ	−	−	−	−	−	−	−	−	+
**MM-177**	M	73	MM	IIIA	D	Gκ	−	−	−	−	+	−	+	−	−
**MM-179**	M	50	MM	IIIA	D	Gλ	−	+	−	−	−	+	−	−	+
**MM-195**	M	62	MM	IIA	D	Gκ	+	−	−	−	+	−	−	−	−
**MM-200**	F	63	MM	IA	D	Aλ	−	−	−	−	−	−	−	−	+
**MM-202**	M	64	MM	IIIA	D	Gκ	−	−	−	−	−	−	+	na	+
**MM-206**	F	73	MM	IIA	D	Gκ	+	−	−	−	+	−	−	+	−
**MM-207**	F	68	MM	IA	D	Aκ	−	−	−	−	+	−	+	−	−
**MM-208**	M	74	MM	IIA	D	Aλ	+	−	−	−	−	−	−	−	−
**MM-209**	F	65	MM	IIA	D	Gλ	−	−	−	−	−	−	−	−	+
**MM-210**	M	65	MM	IIB	D	Aλ	−	−	−	−	−	−	−	−	−
**MM-212**	F	55	MM	IIIA	D	Gκ	−	+	−	−	−	−	−	−	−
**MM-213**	M	66	MM	IIIA	D	λ	−	+	−	−	+	−	−	−	−
**MM-219**	M	73	MM	IIIA	D	Aλ	−	−	−	−	+	−	−	−	+
**MM-224**	F	52	MM	IIIA	D	Gκ	−	−	+	−	−	−	+	−	−
**MM-229**	M	75	MM	IIA	D	Gκ	−	−	−	−	−	−	−	−	−
**MM-238**	M	58	MM	IIB	D	Gκ	−	−	−	−	+	−	+	−	−
**MM-239**	F	72	MM	IIA	D	Aκ	−	−	−	−	+	−	−	−	+

(*Continued*)

Sample	Sex	Age	Disease	Stage[Δ]	Phase[‡]	PP[†]	t(4;14)	t(11;14)	t(14;16)	t(14;20)	del(13)*	del(17p)*	1q gain*	1p loss*	HD°
**MM-240**	M	70	MM	IA	D	Gλ	–	–	–	–	–	–	–	–	+
**MM-241**	M	54	MM	IIA	D	Gκ	–	–	–	–	–	–	–	–	+
**MM-242**	M	69	MM	IIIA	D	Aκ	–	–	–	–	+	–	–	–	+
**MM-243**	F	68	MM	IIA	D	Gκ	–	–	–	–	–	–	+	–	+
**MM-246**	M	71	MM	IIIA	D	Aλ	–	+	–	–	–	–	–	–	–
**MM-252**	F	77	MM	IIA	D	Aλ	–	+	–	–	–	–	+	–	–
**MM-256**	M	58	MM	IIIA	D	Aκ	–	–	+	–	+	–	–	–	–
**MM-261**	F	66	MM	IIB	D	Gκ	–	–	–	–	+	–	+	–	+
**MM-262**	M	74	MM	IA	D	λ	–	–	–	–	–	–	+	–	–
**MM-263**	F	65	MM	IIB	D	κ	–	–	–	–	+	–	+	–	–
**MM-267**	M	74	MM	IIIA	D	Gλ	–	–	–	–	+	–	–	–	+
**MM-268**	M	77	MM	IIIB	D	Gκ	–	–	–	–	–	–	–	–	–
**MM-269**	F	67	MM	IIA	D	Gκ	–	–	–	–	+	–	–	–	+
**MM-271**	M	76	MM	IA	D	Gκ	–	–	–	–	+	–	–	–	–
**MM-274**	M	59	MM	IIA	D	Gκ	+	–	–	–	–	–	–	–	–
**MM-276**	F	70	MM	IIB	D	Gκ	+	–	–	–	+	–	–	–	–
**MM-278**	M	73	MM	IIA	D	Gλ	–	–	–	–	–	–	–	+	+
**MM-279**	F	71	MM	IIIA	D	Gλ	–	–	–	na	+	–	+	–	na
**MM-280**	M	62	MM	IIIA	D	Gλ	–	+	–	–	–	–	–	–	–
**MM-281**	F	77	MM	IIA	D	Gκ	–	–	–	–	–	–	–	–	–
**MM-282**	M	66	MM	IIIA	D	Gκ	–	–	–	–	–	–	+	–	+
**MM-284**	M	49	MM	IIIA	D	κ	–	+	–	–	na	–	–	–	–
**MM-286**	F	71	MM	IIA	D	Gκ	–	–	–	–	+	–	–	–	–
**MM-295**	F	74	MM	IIIA	D	λ	–	–	–	–	+	–	na	na	na
**MM-300**	M	65	MM	IIB	D	Gκ	–	–	–	–	–	–	na	na	na
**MM-301**	F	72	MM	IIA	D	Aλ	–	–	–	–	–	–	+	–	+
**MM-302**	M	65	MM	IA	D	Aκ	–	–	–	–	–	–	+	–	+

(Continued)

Sample	*Sex*	*Age*	*Disease*	*Stage*[Δ]	*Phase*[‡]	*PP*[†]	*t(4;14)*	*t(11;14)*	*t(14;16)*	*t(14;20)*	*del(13)**	*del(17p)**	*1q gain**	*1p loss**	*HD°*
**MM-308**	M	58	MM	IA	D	Aκ	−	−	−	−	−	−	−	−	−
**MM-313**	M	66	MM	IIIA	D	Gλ	−	+	−	−	−	−	−	−	−
**MM-310**	M	67	MM	IIIA	D	Gλ	−	+	−	−	−	−	−	−	−
**MM-314**	F	70	MM	IIIA	D	Aλ	−	+	−	−	−	−	+	−	−
**MM-317**	M	56	MM	IIA	D	Gλ	−	+	−	−	−	−	+	−	−
**MM-321**	M	63	MM	IIIA	D	Gκ	−	−	−	−	+	−	−	−	+
**MM-327**	F	62	MM	IIA	D	absent	−	−	−	−	+	−	+	−	+
**MM-330**	F	61	MM	IIIA	D	Aκ	+	−	−	−	+	−	+	−	−
**MM-334**	F	45	MM	IA	D	Gλ	−	−	−	−	+	+	+	+	+
**MM-335**	F	68	MM	IIA	D	Aκ	−	−	+	−	+	−	−	−	−
**MM-340**	M	46	MM	IIIA	D	Gλ	−	+	−	−	+	−	−	−	−
**MM-341**	M	65	MM	IIIA	D	Gκ	−	−	−	−	−	−	+	−	−
**MM-343**	M	74	MM	IIIA	D	Aλ	−	+	−	−	−	−	−	−	−
**MM-351**	M	na	MM	IIA	D	Aκ	−	−	−	−	−	−	−	−	−
**MM-362**	F	80	MM	IIA	D	Gλ	−	−	−	−	+	+	+	−	−
**MM-372**	M	54	MM	IA	D	Gκ	−	+	−	−	−	−	+	−	−
**MM-375**	F	78	MM	IA	D	Gκ	+	−	−	−	−	+	−	−	−
**MM-381**	F	69	MM	IA	D	Gκ	−	−	−	na	−	−	−	−	na
**MM-382**	M	85	MM	IIA	D	Gκ	−	−	−	−	+	+	na	na	na
**MM-385**	F	76	MM	IIIA	D	Gλ	−	−	−	−	−	−	−	−	+
**MM-386**	F	76	MM	IIA	D	Aλ	−	na	na	na	−	−	na	na	na
**MM-387**	F	44	MM	IIIA	D	Gκ	−	−	−	−	−	−	+	−	+
**MM-392**	F	53	MM	IA	D	Gλ	−	−	−	−	+	−	+	−	+
**MM-398**	F	65	MM	IIA	D	Gκ	−	+	−	−	+	−	+	−	−
**MM-402**	M	67	MM	IIA	D	Gκ	−	+	−	−	+	−	−	−	−
**MM-405**	M	69	MM	IA	D	Gκ	−	+	−	−	−	−	−	−	−
**MM-406**	M	62	MM	IIB	D	Aκ	−	+	−	−	−	−	+	−	na

(Continued)

Sample	Sex	Age	Disease	Stage[Δ]	Phase[‡]	PP[†]	t(4;14)	t(11;14)	t(14;16)	t(14;20)	del(13)*	del(17p)*	1q gain*	1p loss*	HD°
**MM-407**	F	79	MM	IIIA	D	λ	−	−	−	−	+	−	−	−	−
**MM-410**	F	79	MM	IIIA	D	λ	−	−	−	−	−	−	+	−	−
**MM-411**	M	67	MM	IIIA	D	Gλ	−	−	−	−	−	−	−	−	+
**MM-413**	F	51	MM	IIA	D	Gκ	−	−	−	−	+	−	+	−	+
**MM-414**	F	67	MM	IIIA	D	Gλ	−	+	−	−	−	−	−	−	−
**MM-422**	F	74	MM	IIB	D	κ	−	−	−	−	+	−	−	−	−
**MM-423**	F	53	MM	IIA	D	λ	+	−	−	−	+	−	−	−	−
**MM-424**	F	84	MM	IA	D	Gκ	−	−	−	−	+	−	−	+	+
**MM-425**	F	65	MM	IIA	D	Aλ	−	−	−	−	+	−	+	−	−
**MM-428**	F	65	MM	IIA	D	Aλ	−	−	−	−	−	−	+	+	−
**MM-429**	F	63	MM	IIIA	D	Gκ	−	−	−	−	−	−	+	−	+
**MM-430**	na	62	MM	IIIA	D	Gλ	−	−	−	−	−	−	−	−	−
**MM-431**	F	70	MM	IA	D	Gλ	+	−	−	−	+	−	+	−	−
**MM-433**	M	72	MM	IIB	D	κ	−	+	−	−	−	−	−	−	−
**MM-434**	F	53	MM	IIA	D	κ	−	−	−	−	+	−	−	+	−
**MM-435**	na	42	MM	IIIA	D	Gλ	−	−	−	−	−	−	−	−	+
**MM-437**	F	72	MM	IIB	D	λ	−	−	−	−	+	−	+	−	−
**MM-440**	na	61	MM	IIA	D	Aκ	−	−	−	−	−	−	−	+	+
**MM-441**	na	na	MM	IA	D	Gλ	−	−	−	−	−	−	−	−	−
**MM-442**	F	65	sPCL	/	D	Gκ	−	−	−	−	+	−	+	+	−
**MM-445**	na	65	MM	IIA	D	κ	−	+	−	−	+	−	+	−	−
**MM-446**	na	52	MM	IIIA	D	Gλ	−	−	−	−	−	−	+	−	+
**MM-447**	na	57	MM	IIA	D	Gκ	−	+	−	−	−	−	+	−	−
**MM-448**	na	59	MM	IIA	D	Aκ	−	−	−	−	+	−	+	−	−
**MM-449**	na	54	MM	IIA	D	Gκ	−	−	−	−	+	−	−	+	−
**MM-464**	M	75	MM	IIIA	D	Gλ	−	−	−	−	+	−	+	−	−
**PCL-001**	F	51	pPCL	/	D	κ	−	−	−	+	+	−	na	na	na

(*Continued*)

Sample	Sex	Age	Disease	Stage[Δ]	Phase[‡]	PP[†]	t(4;14)	t(11;14)	t(14;16)	t(14;20)	del(13)*	del(17p)*	1q gain*	1p loss*	HD°
**PCL-002**	F	69	sPCL	/	R	λ	–	+	–	–	+	na	–	na	na
**PCL-004**	M	72	pPCL	/	D	Gκ	–	–	+	–	–	–	–	–	–
**PCL-005**	M	76	sPCL	/	R	Aκ	–	+	–	–	–	–	–	–	–
**PCL-008**	M	57	pPCL	/	D	Gλ	–	–	+	–	+	–	–	na	na
**PCL-009**	F	77	sPCL	/	R	Gκ	+	–	–	–	+	–	+	–	–
**PCL-011**	M	76	sPCL	/	R	Gκ	–	–	+	–	+	+	+	–	–
**PCL-012**	F	62	sPCL	/	R	Aκ	–	–	+	–	–	+	–	–	–
**PCL-014**	M	72	pPCL	/	D	λ	–	+	–	–	+	+	–	+	–
**PCL-015**	M	78	pPCL	/	D	κ	–	–	+	–	+	–	+	–	–
**PCL-016**	F	57	pPCL	/	D	Gκ	–	–	+	–	+	–	+	+	–
**PCL-017**	F	68	pPCL	/	D	Gκ	–	–	+	–	+	+	+	+	–
**PCL-018**	F	59	pPCL	/	D	κ	–	+	–	–	+	+	–	–	–
**PCL-019**	F	67	pPCL	/	D	Mκ	–	–	+	–	+	+	+	+	–
**PCL-020**	F	79	pPCL	/	D	Gλ	–	–	–	–	–	–	+	–	–
**PCL-021**	M	48	pPCL	/	D	Gλ	+	–	–	–	+	–	+	–	–
**PCL-023**	M	60	pPCL	/	D	Gκ	–	–	+	–	+	+	+	+	–
**PCL-026**	F	59	pPCL	/	D	Gκ	–	–	+	–	+	–	+	–	–
**PCL-027**	M	65	pPCL	/	D	λ	–	+	–	–	–	+	–	–	–
**PCL-028**	F	57	pPCL	/	D	κ	–	+	–	–	+	–	–	–	–
**PCL-029**	M	51	pPCL	/	D	Aλ	–	–	+	–	–	–	–	–	–
**PCL-030**	F	52	pPCL	/	D	κ	–	–	–	+	+	+	–	–	–
**PCL-031**	F	59	sPCL	/	D	Gλ	–	–	+	–	–	+	+	–	–
**PCL-032**	M	51	pPCL	/	D	Gκ	+	–	–	–	+	–	+	–	–
**PCL-035**	F	76	pPCL	/	D	κ	–	+	–	–	–	–	–	–	–
**PCL-036**	M	72	pPCL	/	D	Gκ	–	+	–	–	+	–	–	–	–
**PCL-037**	M	72	pPCL	/	D	Aλ	–	+	–	–	+	–	–	–	–
**PCL-038**	M	57	pPCL	/	D	Gκ	–	–	–	–	+	–	+	+	+

(Continued)

Sample	*Sex*	*Age*	*Disease*	*Stage*[Δ]	*Phase*[‡]	*PP*[†]	*t(4;14)*	*t(11;14)*	*t(14;16)*	*t(14;20)*	*del(13)**	*del(17p)**	*1q gain**	*1p loss**	*HD°*
**PCL-039**	M	54	sPCL	/	D	Aκ	–	–	–	–	–	+	–	+	–
**PCL-041**	M	na	sPCL	/	D	Gκ	–	na	na	na	–	–	na	na	na
**PCL-042**	F	69	sPCL	/	D	Gλ	–	+	–	–	–	–	–	–	–
**PCL-043**	F	68	pPCL	/	D	Gλ	–	+	–	–	–	–	–	–	–
**PCL-046**	F	50	pPCL	/	D	κ	–	–	–	–	+	–	+	–	+

[Δ] The Durie clinical staging system was adopted; [‡]D: Diagnosis; R: relapse;
[†] Paraprotein; *del(13), del(17), 1p loss and 1q gain were determined by FISH.
[°] HD: presence of the hyperdiploid status on the basis of FISH evaluation criteria. *na*: not available.

Supplementary Table S6. Genome-specific primers for amplicon library preparation

Exon	Amplicon localization (hg19, chromosome 13)	Primer FWD	Primer REV
**1**	73355681–73356065	5′- GAAAGGGAAGAACCTCCGGG-3′	5′- TCCCTGTCATACCCCCTTGT-3′
**2**	73354916–73355271	5′- GCTTCTTGGCTTAACTTATTCAGTG-3′	5′- ACAGGAACCCTCTCCCGAA-3′
**3**	73352200–73352586	5′- TGCTAAGAGTTTTCACATATCCTTG-3′	5′- TCACATGAAGTTATATAGGACTACGA-3′
**4**	73351452–73351809	5′- AGGCTGTAGTGATGTGTAATTGC-3′	5′- GCTTACCCACCGACATTCC-3′
**10**	73346062–73346470	5′- TATGTTGTAGTTGTGCTTTGGAAAT-3′	5′- CAATATGCTTGACTGGGTAAATGTA-3′
**11**	73345868–73346221	5′- TTTGCTTGTTAATTACTCTTGTGAAG-3′	5′- TTCATGGCCGTTTAAGAATC-3′
**12–13**	73345012–73345325	5′- TTATGGCTAAGTAATCTGTGGTTCTA-3′	5′-AAATTAGAGATTAATAGCCATGAAACG-3′
**14**	73342807–73343130	5′- GTAGTGAAAGTAGGAGGACATATTG-3′	5′- GAAGCTAGAAGAAACAGGAGTCT-3′
**15**	73339975–73340297	5′- ACACTTGCTGTAGTCATTGTCTT-3′	5′- GCAAGCCAAATAAAGTAGAAATCAT-3′
**16**	73337539–73337917	5′- GCGGAGTAACTGAGAGATGAAAG-3′	5′- CAGGTAGATCAAAACACAAATAGATG-3′
**17**	73335921–73336221	5′- GCCGAATCTCCTACTTTTCCA-3′	5′- CCAAAAGCCGATGAACAATGA-3′
**18**	73335757–73336105	5′- CTTAGCGTCTCCAATATACACACA-3′	5′- CTAGCAGTATCGACAAAAGGCA-3′

Supplementary Table S7. Sequence-specific exonic PCR primers

Primer FWD	Primer REV
5′- CCGGGGTTAGGCGTATTCTA -3′	5′- AAGTGAGAAATCGCAGTGCC -3′
5′- TACTTGCTGCCCGACACTAA -3′	5′- GGAATACCAGCTTTCACTTGTG -3′
5′- ATGTTCCCCATCAGCCTTTT -3′	5′- AAAATGTGACGTGGACAGGC -3′
5′- GGGAAATGAATCACAATGCTGA -3′	5′- TCTGAACATGCTCTGCTTCG -3′
5′- AGTCTTTGGATCAGGCCGAA -3′	5′- GGCTATTGGGGCTGACTGTA -3′
5′- ACTATGGCTTAGCGTCTCCA -3′	5′- AACGTGCATCAGTGGCTTTT-3′
